# Valorization of Fish Waste: Isolation and Characterization of Acid- and Pepsin-Soluble Collagen from the Scales of Mediterranean Fish and Fabrication of Collagen-Based Nanofibrous Scaffolds

**DOI:** 10.3390/md20110664

**Published:** 2022-10-25

**Authors:** Leto-Aikaterini Tziveleka, Stefanos Kikionis, Labros Karkatzoulis, Kostas Bethanis, Vassilios Roussis, Efstathia Ioannou

**Affiliations:** 1 Section of Pharmacognosy and Chemistry of Natural Products, Department of Pharmacy, National and Kapodistrian University of Athens, Panepistimiopolis Zografou, 15771 Athens, Greece; 2 Laboratory of Physics, Department of Biotechnology, Agricultural University of Athens, 11855 Athens, Greece

**Keywords:** fish scales, marine collagen, acid-soluble collagen, pepsin-soluble collagen, nanofibers

## Abstract

In search of alternative and sustainable sources of collagenous materials for biomedical applications, the scales of five Mediterranean fish species—fished in high tonnage in the Mediterranean region since they represent popular choices for the local diet—as well as those of the Atlantic salmon for comparison purposes, were comparatively studied for their acid- and pepsin-soluble collagen content. Fish scales that currently represent a discarded biomass of no value could be efficiently exploited for the production of a high added-value biomaterial. The isolated collagenous materials, which showed the typical electrophoretic patterns of type I collagen, were morphologically and physicochemically characterized. Using scanning electron microscopy the fibrous morphology of the isolated collagens was confirmed, while the hydroxyproline content, in conjunction with infrared spectroscopy and X-ray diffraction studies verified the characteristic for collagen amino acid profile and its secondary structure. The acid- and pepsin-soluble collagens isolated from the fish scales were blended with the bioactive sulfated marine polysaccharide ulvan and polyethylene oxide and electrospun to afford nanofibrous scaffolds that could find applications in the biomedical sector.

## 1. Introduction

Collagen is the most abundant extracellular matrix (ECM) protein of all eukaryotic tissues, playing a decisive role in structural and biological integrity maintenance [1]. Occurring in both invertebrate and vertebrate organisms, collagen exists in at least 29 different types, commonly distributed in skin, bones, cartilage, tendons, cornea, ligaments, and other vertebrate organs [1,2,3,4]. Collagen’s unique structure is attributed to the entanglement of its three polypeptide α-chains forming a characteristic right-handed triple helix that self-assembles into insoluble fibers of great tensile strength. The main building blocks of these polypeptides are glycine, proline, and hydroxyproline, all being essential for the formation of hydrogen bonds and the stabilization of the helical configuration.

Collagen finds a plethora of applications, primarily in the pharmaceutical, cosmeceutical, nutraceutical, and biomedical sectors [4,5,6,7,8]. Its inherent biocompatibility and biodegradability, as well as its low immunogenicity, renders it ideal material for tissue engineering applications [1,9]. Most notably, type I collagen, the major fibrillar component of many tissues, such as skin, bone, tendon, and cartilage, is commonly used in tissue regeneration and other biomedical applications [10]. Particularly, electrospun collagen-based nanofibers are considered as a promising type of biomaterial since fabricated biomimetic scaffolds resemble the architecture of native human tissues [11,12]. Since collagen nanofibers possess poor mechanical properties, blending with synthetic polymers is commonly used for improvement of their mechanical strength without any adverse effect on the bioactivity. In this perspective, collagen blends with synthetic polymers, such as polyethylene oxide (PEO) [13], polycaprolactone (PCL) [14], polylactic acid (PLA) [15,16], poly(L-lactic acid-co-ε-caprolactone) (PLCL) [17], and poly(vinyl) alcohol (PVA) [18], have been prepared for tissue engineering applications.

Traditionally, the major source of commercial collagen has been terrestrial mammals, especially cattle and pigs. Nowadays, the use of bovine and porcine collagen is being questioned, due to health-related, ethical, and religious considerations [19]. Hence, environmentally friendly, sustainable, and safer alternative sources of collagen are sought.

Currently, collagen of marine origin is considered as a potential alternative [20,21,22], especially since the employment of recombinant technology is restricted due to its high cost [23]. In addition to being safer, several reports have shown that marine collagen and its presence in biomaterials promotes cell adhesion, differentiation, and growth, as well as wound-healing [24]. Marine collagen can be extracted from fish processing by-products, such as skin, bones, and scales [25], ensuring the sustainable exploitation of marine resources through the valorization of residues, while reducing the related environmental footprint [20,22,26]. Particularly, fish scales, a by-product of the fish canning industry as well as of fish markets, could be exploited as a source of relatively high purity collagen instead of being discarded as waste during processing, as they contain high amounts of type I collagen but low amounts of lipids. Effective waste management could turn the discards into assets, increasing economic profits for the fishermen and the fish industry.

In the present study, we report the isolation and characterization of acid- and pepsin-soluble collagens isolated for the first time from the scales of five Mediterranean fish species that are fished in high tonnage in the Mediterranean region since they represent popular choices for the local diet, as well as from the scales of the Atlantic salmon, isolated and analyzed for comparison. The morphological, physicochemical, and biochemical characteristics of these biomacromolecules are analyzed and discussed regarding their potential use in the biomedical sector. Aiming at the development of collagen-based nanofibrous scaffolds that could find applications in the biomedical field, the marine sulfated polysaccharide ulvan was chosen as an additional bioactive component due to the wide range of bioactivities it exhibits [27], including its osteoinductive capacity [28,29]. Therefore, the isolated collagens were co-blended with ulvan and PEO and the fabricated composite nanofibers were morphologically and physicochemically characterized.

## 2. Results and Discussion

### 2.1. Isolation and Characterization of Acid- and Pepsin-Soluble Collagen

Acid- and pepsin-soluble collagens from the scales of five Mediterranean fish species, namely dusky grouper (*Epinephelus marginatus*), red mullet (*Mullus barbatus*), common seabream (*Pagrus pagrus*), gilt-head bream (*Sparus aurata*), and shi drum (*Umbrina cirrosa*), that are among the most commonly fished species in hundreds of tons in the Mediterranean Sea since they represent popular choices for the local diet, as well as those of the Atlantic salmon (*Salmo salar*) for comparison, were isolated and characterized.

Two washing steps were employed during the procedure followed for the isolation of collagen from the fish scales. The first one, salt washing, was employed so as to remove non-collagenous proteins, while the second one, EDTA washing, resulted in the decalcification of the scales. The yield for the ASCs ranged between 0.39 and 1.13% (per dry weight of scales), with the highest yield observed for *S. aurata* and the lowest for *E. marginatus* (Table 1). The yields for PSCs, attained after cleavage of the cross-linked telopeptide region of collagens, were 1.4 to 4 times higher than those for ASCs, ranging from 1.04 to 2.03% (per dry weight of scales), observed for *M. barbatus* and *S. salar*, respectively (Table 1).

A number of studies have reported similar trends in the yields of ASC and PSC obtained from scales of the fish *Cyprinus carpio* [30], *Miichthys miiuy* [31], *Parupeneus heptacanthus* [32], *Sardinella longiceps* [33], and *Sciaenops ocellatus* [34], indicating that the collagen’s telopeptide region is highly cross-linked in fish scales [35]. Similarly, higher yields have been reported for PSCs isolated from the skin of Amur sturgeon [36] and from the cartilages of shark [37], as compared with the ASCs isolated from the respective species and tissues. Therefore, it can be concluded that the collagens isolated from *E. marginatus* and *U. cirrosa* (with PSC-to-ASC ratios equal to 4) are the most intensely cross-linked among the studied species.

The hydroxyproline content of the ASCs and PSCs isolated from the fish scales examined ranged from 35.1 to 52.5 and from 39.6 to 58.6 mg/g dry sample, respectively (Table 1). Overall, statistically significant differences were detected among some of the different species analyzed, especially in their PSCs, but not among the different type of collagens (ASCs and PSCs) examined. Only in the case of *S. salar* were statistically significant differences detected among its ASC and PSC. In particular, the PSC contained more hydroxyproline than ASC, pointing to the existence of exceptionally hydroxyproline-poor telopeptides. These results are in accordance with previous work of Kittiphattanabawon and colleagues who reported statistically significant variations among the sources (bone, skin, etc.) and the species, but not amidst acid- and pepsin-soluble collagens [37,38]. Therefore, it can be concluded that telopeptides are of relatively consistent sequence but not enriched in hydroxyproline. It is well established that hydroxyproline contents vary with species, environment, and body temperature of fish [39]. Therefore, differences in the hydroxyproline content of *S. salar* can be expected due to the lower environmental temperature in which this species lives.

**Figure 1:**
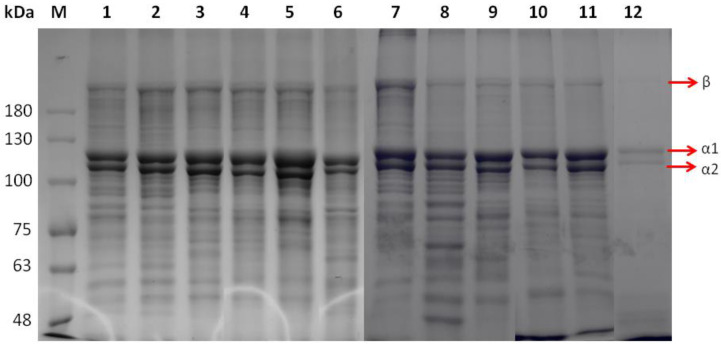
SDS-PAGE patterns of ASCs (lanes 1–6) and PSCs (lanes 7–12) from *E. marginatus* (lanes 1, 7), *M. barbatus* (lanes 2, 8), *P. pagrus* (lanes 3, 9), *S. aurata* (lanes 4, 10), *U. cirrosa* (lanes 5, 11), and *S. salar* (lanes 6, 12). Protein markers are depicted as M.

ASCs isolated from the different species were analyzed under reducing conditions with SDS–PAGE (Figure 1, lanes 1–6). The acquired protein patterns showed that all ASCs consisted of two different α chains (α1 and α2, ~128 and 118 kDa, respectively) as well as a cross-linked β component (~190 kDa, dimers), while no significant differences in patterns among the different species were observed. The subunit composition and electrophoretic mobility of ASCs indicated type I collagen, which contains two identical α1 chains and one α2 chain in the molecular form of [2α1(I)α2(I)], as the major collagen type prevalent in the investigated fish scales. Specifically, all the ASCs consisted of two different α chains (α1 and α2), the intensity of α1 being stronger than that of α2 (α1-to-α2 ratio up to 1.9). Type I collagen has been previously reported to be the primary collagen found in fish scales [40,41,42,43]. Type I collagens from lizard fish, spotted golden goatfish, and tilapia scales have been reported to contain α1 and α2 chains with MW of 120, 117, and 132, and 110, 108, and 121 kDa, respectively [32,42,43]. The MW of α chains is usually estimated by employing globular protein standards that might lead to overestimations due to differences between globular and collagenous proteins with high content of relatively small amino acid residues (Gly and Ala) [44]. The differences observed in MW have been associated with differences in the amino acid composition.

PSCs were also shown to contain at least two different α chains (Figure 1, lanes 7–12). PSCs were composed of both α1 and α2 chains (~125 and 116 kDa, respectively), with an α1-to-α2 ratio reaching the value of 1.7. Such collagens have been previously reported to consist of two α1 chains and a single α2 chain [34]. The presence of peptides with MW below 100 kDa was noticeable in most of the PSC samples. This may result from the enzyme used during the PSC extraction process that induced cleavage to the protein chains [45].

β- and γ-components (dimers and trimers, respectively) were only faintly observed in all the ASCs and PSCs isolated. It is well documented that intensive intra- and intermolecular covalent cross-linking results in the stabilization of collagen fibrils against proteolytic degradation and promotes the desired tensile strength. However, the cross-linking of adjacent chains into dimers (β-chains) or trimers (γ-chains) may hinder the collagen folding to a triple-helix conformation by interfering with the degree of freedom of the individual monomers [24].

**Figure 2:**
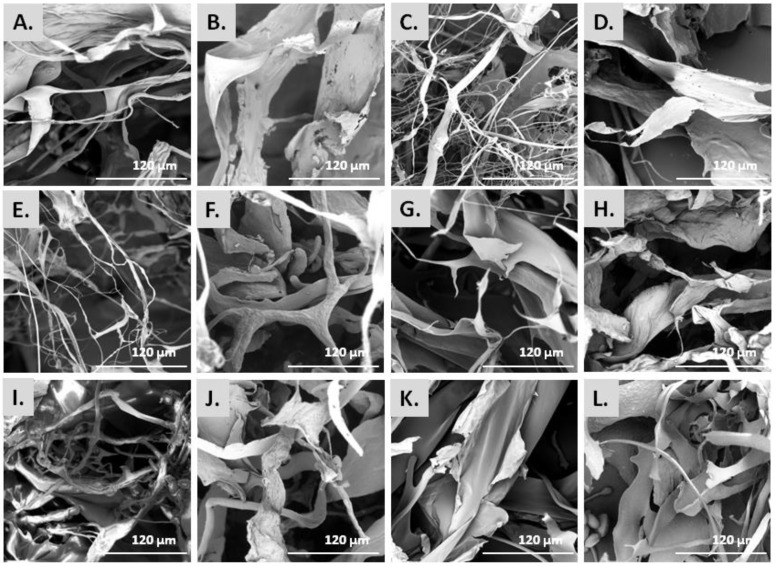
SEM micrographs [scale bar = 120 µm (×1000)] of ASCs and PSCs from *E. marginatus* (**A**,**B**), *M. barbatus* (**C**,**D**), *P. pagrus* (**E**,**F**), *S. aurata* (**G**,**H**) *U. cirrosa* (**I**,**J**), and *S. salar* (**K**,**L**).

The morphology of the isolated collagenous materials was examined by SEM. Figure 2 depicts the microstructure of the ASCs and PSCs isolated from the scales of the different fish species, as observed by SEM analysis. Specifically, most ASCs were observed as smoothly wrinkled and folded sheets and threads of various diameters, which is the combination of several collagen fibrils and fibers that are bundled together to form a fibril network and a densely pleated sheet-like structure (Figure 2A,C,E,I). In the case of ASCs isolated from *S. aurata* (Figure 2G) and *S. salar* (Figure 2K), mostly smooth pleating of the sheets was visible which appeared as very thin and soft at a magnification of ×1000. In addition, the PSCs isolated from the scales of all studied species presented the typical striation and dense sheet-like appearance of collagen fibers (Figure 2B,D,F,H,J,L). In particular, PSCs displayed an irregular, multi-layered, aggregated fibrous mesh work appearance, possibly due to the enzyme treatment.

Overall, the microscopically observed structures (Figure 2) were similar to those already reported for collagen isolated from the skin and bone of Spanish mackerel [45], the skins of Nile tilapia [46] and Amur sturgeon [36], the scales of croceine and redlip croakers [47], and marine finfish scales [33]. Hence, the complex porous microscopic appearance of all studied ASCs and PSCs indicated that they could be utilized as hydrating agents, in wound dressings, as matrices for cell proliferation, or for other tissue engineering applications [20,21].

UV absorbance in proteins arises primarily from their peptide bonds and their side chains. Previous reports [31,48,49] indicated that in the case of collagen, maximum absorption is observed in the range 210–240 nm, which is mainly attributed to peptide bond absorptions by n→π* transitions of the groups of C=O, COOH, and CONH_2_ in the polypeptide chains of collagen [50,51]. The maximum absorption peaks of the obtained ASCs and PSCs were observed at 230 and 232 nm, respectively (Figures S1–S6). The weak absorbance at 250–280 nm shows that the fish scale acid- and pepsin-soluble collagens have low concentrations of aromatic amino acids, such as phenylalanine, tryptophan, and tyrosine. The absorption peaks observed are in agreement with previous findings reported for sardine (*S. longiceps*) fish scales (230 and 232 nm for ASC and PSC, respectively) [33] and for pepsin-soluble type I collagen from the scales of red drum fish (*S. ocellatus*) (230 nm) [34].

**Figure 3:**
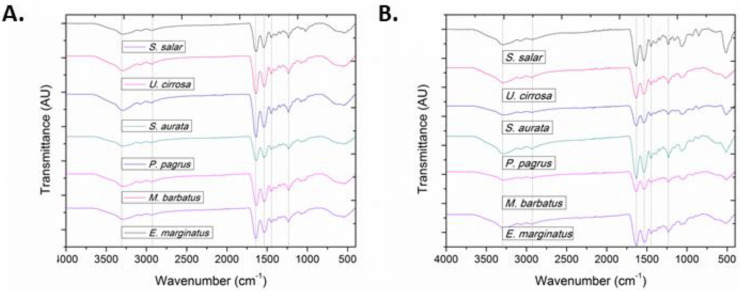
FT-IR spectra of (**A**) ASCs and (**B**) PSCs from *E. marginatus*, *M. barbatus*, *P. pagrus*, *S. aurata*, *U. cirrosa*, and *S. salar*.

All characteristic absorption bands of collagenous materials (amides A and B, as well as amides I, II, and III) were observed in the FT-IR spectra of the isolated ASCs and PSCs (Figure 3) [52,53]. More importantly, all bands in the FT-IR spectra of both ASCs and PSCs isolated from the same species exhibited only slight differences, indicating that enzymatic treatment did not significantly affect their secondary structure (Table S1) [46,54,55,56].

Specifically, the amide A absorption band, associated with the N–H stretching vibrations, was observed at low frequencies (3295 to 3283 cm^−1^), indicative of the involvement of the NH groups in hydrogen bonding [53], since free N–H stretching vibration typically occurs in the range of 3400–3440 cm^−1^. Moreover, the amide B absorption band, attributed to the asymmetrical stretch of CH_2_ and NH_3_^+^, remained rather consistent (~2931 cm^−1^). Only in the case of ASC and PSC isolated from *E. marginatus*, the amide B band peaked at approximately 2920 cm^−1^, possibly due to differences in the lysine content as compared with the other examined species [57].

Amide I, amide II, and amide III bands, indicative of the existence of the triple-helical structure of collagen, are known to be associated to the degree of molecular order [44]. Payne and Veis (1988) reported that the shift of amide I, predominantly, and amide II bands to higher frequencies is associated with increase of the molecular order. Specifically, the amide I band, representing the vibrations of amide carbonyl groups on the polypeptide backbone, is the most intense band in proteins and is considered as a susceptible marker for the monitoring of conformation-dependent spectral changes [58]. Bands at approximately 1630 and 1645 cm^−1^ suggest imide residues and random coils, while bands at approximately 1650 and 1675 cm^−1^ are assigned to helical structures and β-turns, respectively. In all the ASCs and PSCs examined, the amide I component bands absorb primarily at 1644 and 1633 cm^−1^, with a ranging ratio between the two bands indicative of higher hydrogen bonding, reduced intermolecular cross-linking, and decreased molecular order [57,59,60,61,62,63]. Furthermore, the amide II band, attributed to the N–H bending vibration coupled with the C–N stretching vibration, was mostly constant but also shifted to lower frequencies (1541 to 1538 cm^−1^), indicative of the involvement of the N–H groups in hydrogen bonding with the adjacent α-chains. Especially in the case of PSC isolated from *S. salar*, the amide II band peaked at even lower wavenumbers (1532 cm^−1^). The amide III band represents a combination of the N–H bending and the C–N stretching vibration, as well as absorptions arising from wagging vibrations from the CH_2_ groups of the glycine backbone and proline side chains [64], also referred to as the collagen fingerprint [59]. The absorption intensity ratio between the amide III band (1236 to 1232 cm^−1^) and the band at approximately 1450 cm^−1^ was almost 1 for both the ASCs and PSCs isolated from all examined species, indicating that the isolation procedure preserved the triple helical structures [54,57,65]. Specifically, the ratio ranged between 0.94 and 1.0 for the ASCs and between 0.88 and 0.99 for the PSCs. It has been earlier shown that this ratio might be lower when the collagen triple helix is affected by cleavage of telopeptides through pepsin digestion [37].

Conclusively, the triple helical structures of ASCs and PSCs isolated in this study were well maintained, although the molecular structure of collagens from different species had slight variations. In general, ASCs possessed a higher degree of intermolecular cross-links and molecular order in comparison with PSCs.

**Figure 4:**
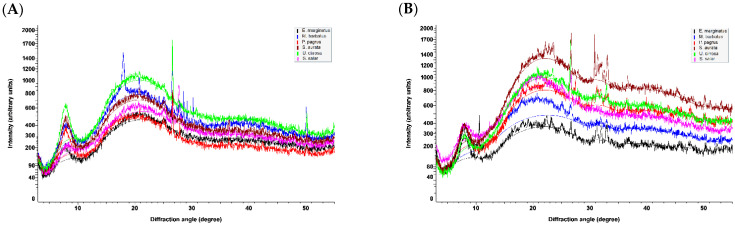
X-ray diffraction diagrams of freeze-dried (**A**) ASCs and (**B**) PSCs from *E. marginatus, M. barbatus, P. pagrus, S. aurata*, *U. cirrosa*, and *S. salar*.

In the X-ray diffraction diagrams obtained for the freeze-dried samples of both ASCs and PSCs (Figure 4), the position of the first sharp peak, indicating the distance between the molecular chains, is found at diffraction angles (2*θ*) that span between 7.74 and 8.20°. By using the Bragg equation *d*(Å) = λ/2 sin *θ*, where λ is the X-ray wavelength (1.5408 Å) and *θ* is the Bragg diffraction angle, the calculated spacing *d* values for the examined samples vary between 10.77 and 11.41 Å. These types of peaks are characteristic of collagen and are associated with its triple-helical structure [41,43,46,66]. The observed spacing values indicate that the collagen molecules in all samples have the characteristic triple-helix structure being undenatured, retaining their native conformation. It is interesting to note the significant difference in the intensity of that peak observed for the ASC and PSC isolated from *U. cirrosa* (Figure S7), indicating a highly ordered structure in the case of ASC which justifies the hypothesis of intensely cross-linked molecules, as has been also predicted by the PSC-to-ASC yield ratio calculated at approx. 4.

A second characteristic of collagen, broad peak, is observed in all diagrams at an approximate 21° diffraction angle. This corresponds to a spacing of approximately 4.2 Å and is attributed to diffuse scattering [66]. Several very intense peaks (e.g., at 2*θ* of approximately 10.5°, 26.5°, 27.8°, and 50.1°) are due to the presence of calcium salts (hydroxyapatite, calcium carbonate, calcium hydroxide, etc.) that remained after the demineralization process. The high crystallinity of these compounds causes an efficient and intense X-ray diffraction despite their small amount in the samples.

Finally, a bundle of peaks from approximately 31.1 to 33.1° diffraction angles is observed in the cases of PSCs obtained from *E. marginatus, P. pagrus, S. aurata*, and *U. cirrosa*. The *d* values (2.87 to 2.71 Å) corresponding to these peaks represent the distance between adjacent amino acid residues along the central axis of the helical structure [43]. The spread of these peaks in these samples and their absence from the remaining samples are due to the fact that the distances between amino acid residues within the helical part of the collagen molecule is not uniform, as the collagen sequence variability translates to deviations from the ideal polyproline II helix [67].

**Figure 5:**
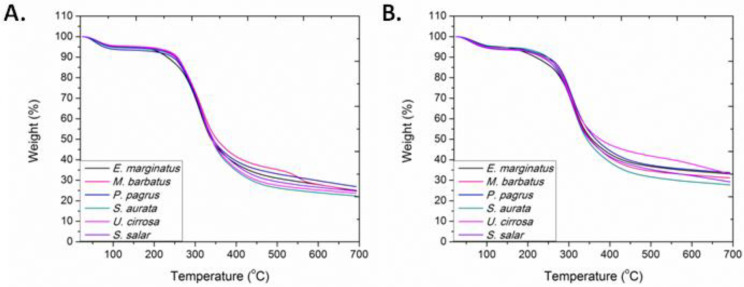
TGA thermogravimetric curves of (**A**) ASCs and (**B**) PSCs isolated from *E. marginatus*, *M. barbatus*, *P. pagrus*, *S. aurata*, *U. cirrosa*, and *S. salar*.

TGA, which provides a quantitative measurement of mass change associated with dehydration, decomposition, and oxidation as a function of time and temperature, was employed for assessing the thermal properties of ASCs and PSCs. Their characteristic thermogravimetric curves are presented in Figure 5. The initial mass loss, credited to the elimination of absorbed and bound water and observed below 100 °C, ranged between 4.3 and 6.1% for the ASCs and between 4.4 and 5.5% for the PSCs. The major decomposition, attributed to the fragmentation of collagen, as related to the main degradation, was observed between 315 and 323 °C for the ASCs and between 303 and 315 °C for the PSCs (Figure S8). In all samples examined, the temperature where the main decomposition occurred was higher for ASCs than PSCs, suggesting the stabilizing role of the collagen’s telopeptides. The total ash content was found to range between 22.4 and 26.9 wt.% in the ASCs and between 25.9 and 33.7 wt.% in the PSCs.

### 2.2. Fabrication and Characterization of Collagen-Based Nanofibrous Scaffolds

Previously, fish scale and skin collagens were combined with nanohydroxyapatite and PLGA to produce collagen/nanohydroxyapatite/PLGA composite nanofibrous membranes via electrospinning for guided bone regeneration [68]. In another approach, a marine collagen/PCL composite nanofibrous scaffold was electrospun for epithelial tissue or lymphoid tissue engineering applications. In addition to facilitating cell adhesion, spreading, protrusions, and proliferation of mouse thymic epithelial reticular cells, this scaffold was proven to stimulate the expression of genes and proteins involved in cell adhesion and T-cell development [69]. Similarly, biomimetic electrospun tilapia fish collagen/bioactive glass/chitosan nanofibers were developed, exhibiting enhanced cell adhesion, cell viability, and induction of expression of osteogenic genes in hPDL cells [70]. In another study, PEO was used as a spinning aid to prepare fish skin collagen–PCL nanofiber membranes via electrospinning. In this case, the diameter of the fibers became fine and smooth as the ratio of the collagen/PCL increased. The obtained nanofibers had good mechanical strength, porosity, and hydrophilicity, and could promote cell adhesion and proliferation [71].

In this study, aiming to prepare collagen-based nanofibers that could find applications in the biomedical sector, the sulfated marine polysaccharide ulvan was selected as an additional bioactive component due to its well-documented biological activities [27], including its osteoinductive capacity, highlighting its potential for tissue engineering applications [28,29]. More recently, nanofibrous matrices based on ulvan and marine gelatin were prepared and evaluated on a second-degree burn model, promoting fast wound contraction during the early stages of the burn-wound-healing process, suppressing inflammation, and achieving uniform wound closure [72]. In addition, the biocompatible PEO was added to the spinning solutions to act as a polymer carrier enhancing the electrospinnability of the polymeric blends.

**Figure 6:**
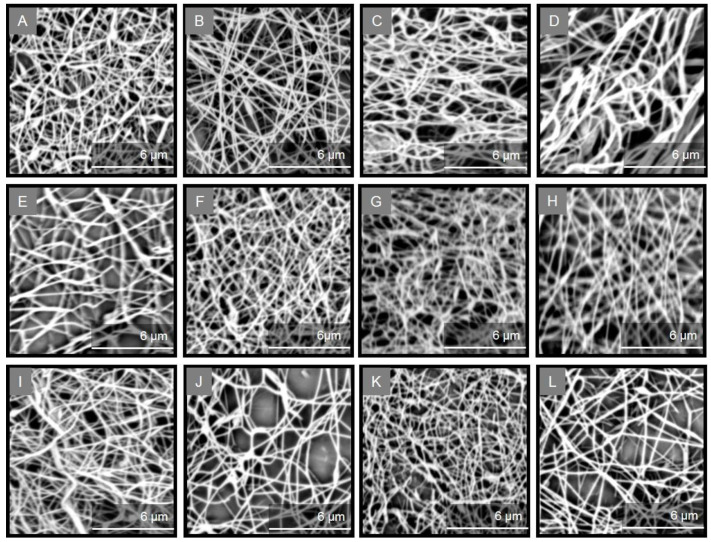
SEM images [scale bar = 6 µm (×20,000)] of the collagen/ulvan/PEO nanofibrous scaffolds containing ASCs and PSCs isolated from *E. marginatus* (**A**,**B**), *M. barbatus* (**C**,**D**), *P. pagrus* (**E**,**F**), *S. aurata* (**G**,**H**), *U. cirrosa* (**I**,**J**), and *S. salar* (**K**,**L**).

Uniform, bead-free fibers were obtained for all ASCs and PSCs by fine-tuning the electrospinning parameters, as evidenced from the SEM images (Figure 6). The non-woven scaffolds revealed a similar fibrous morphology and, in all cases, a well-formed fibrous network was observed with smooth fibers of cylindrical shape and diameter sizes in the nanometer scale (Table 2). ASC-containing fibers were approximately 120 to 640 nm in size, with the average diameter ranging from 287 to 348 nm. Similarly, PSC-containing fibers were approximately 54 to 800 nm in size, with the average diameter ranging from 273 to 364 nm.

**Figure 7:**
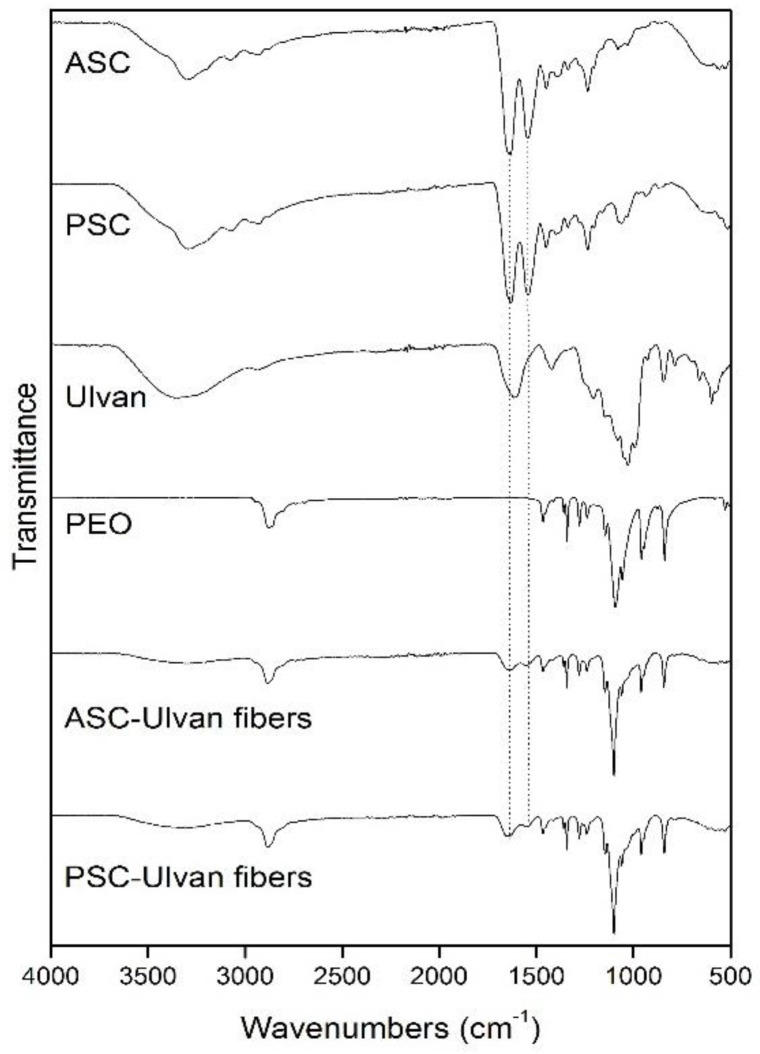
FT-IR spectra of ASC, PSC, ulvan, PEO, characteristic ASC/ulvan/PEO, and PSC/ulvan/PEO composite fibers.

All nanofibrous scaffolds exhibited similar FT-IR spectra revealing the characteristic absorption bands of their ingredients, slightly shifted in comparison with those observed in the FT-IR spectra of the individual components due to their interactions within the polymer matrix (Figure 7). The incorporation of collagen and ulvan into the fibrous scaffolds was evident by the characteristic broad absorption band at approximately 3310 cm^−1^ attributed to the overlapping –OH and –NH (amide A) signals of the saccharide and amide structures. The characteristic bands of amide I and amide II, overlapping with the band of the –C=O carboxylic groups of ulvan, were observed as broad absorption bands at 1641 and 1543 cm^−1^, respectively, verifying the structural integrity of collagen after the electrospinning process. Lastly, the PEO absorption bands were evident at 2883 cm^−1^ (–CH_2_ stretching vibration) and at 1101 cm^−1^ (C–O–C stretching vibration).

The developed marine collagen/ulvan-based scaffolds combining the biological properties of these two marine biopolymers in the form of non-woven nanofibrous patches that offer several advantages, such as high surface-to-volume ratio, porosity, and morphology mimicking the ECM, could represent ideal biomaterials for biomedical applications.

## 3. Materials and Methods

### 3.1. Chemicals

EDTA was purchased from Penta chemicals unlimited (Livingston, NJ, USA). Tris(hydroxymethyl)aminomethane was purchased from Mallinckrodt (Dublin, Ireland), Na_2_HPO_4_ and CH_3_COOH were purchased from Merck (Darmstadt, Germany), and NaCl was purchased from Carlo Erba (Barcelona, Spain). PEO (MW 8,000,000), pepsin from porcine gastric mucosa (≥250 units/mg solid), and hydroxyproline assay kit were purchased from Sigma-Aldrich (Darmstadt, Germany). BlueStar prestained protein marker was from Nippon Genetics Europe GmbH (Düren, Germany). The extraction of ulvan from the green alga *Ulva rigida* collected from the island of Kefalonia, Greece, and its chemical analysis (MW distribution centered at approx. 1100 kDa; 48.4% sulfate, 39.6% carbohydrates; among carbohydrates, uronic acids, and rhamnose represented 17.8% and 25.9%, respectively) were performed as previously described [73].

### 3.2. Extraction of Collagen from Fish Scales

Fish scales, provided by the Greek Central Market and Fishery Organizations (OKAA SA), were washed thoroughly with tap water and used for the isolation of ASC and PSC. Collagens were obtained following previously published protocols after modification [36,40,42]. All steps were performed at 8 °C, unless otherwise stated, to avoid collagen chain fragmentation [74].

#### 3.2.1. Non-Collagenous Protein Removal and Demineralization

Initially, fish scales were immersed in 20% NaCl solution with a residue-to-solvent ratio of 1:10 (*w/v*) for 48 h under stirring to remove non-collagenous proteins. The NaCl solution was replaced every 12 h. Following filtration through a 540-mesh filter, the scales were thoroughly washed twice with distilled H_2_O. Demineralization was achieved through stirring of the scales in 0.5 M EDTA (pH 7.4) with a residue-to-solvent ratio of 1:10 (*w/v*) for 72 h. The EDTA solution was replaced every 24 h. Following filtration through a 540-mesh filter, the scales were thoroughly washed three times with distilled H_2_O.

#### 3.2.2. Isolation of Acid-Soluble Collagen

Demineralized fish scales were treated three times with 0.5 M CH_3_COOH (pH 2.5) with a residue-to-solvent ratio of 1:10 (*w/v*) over a period of 72 h under stirring. Subsequently, the insoluble residue (used subsequently for the isolation of pepsin-soluble collagen) was filtered out, NaCl was added to the combined filtrates to a final concentration of 0.9 M to induce salting out of collagen, and the solution was kept at 8 °C for 24 h. The resulting suspension was centrifuged at 10,000× g for 1 h, and the precipitate was dissolved in the minimum volume of 0.5 M CH_3_COOH. The final solution was then dialyzed using a dialysis membrane (MW cut-off range 12–14 kDa) against 0.1 M CH_3_COOH and distilled H_2_O. The resulting dialysate was lyophilized (CoolSafe, Scanvac, LaboGene A/S, Allerød, Denmark), and is hereafter referred to as ‘‘acid-soluble collagen, ASC’’. The extraction yield of ASC was calculated based on the dry weight of the freeze-dried collagen, as:

Yield (%) = (*M*/*M*_0_) × 100

where *M* is the weight of freeze-dried collagen (g), and *M*_0_ is the weight of dry scales used (g).

#### 3.2.3. Isolation of Pepsin-Soluble Collagen

The non-dissolved residue obtained after acid extraction was suspended in 0.5 M CH_3_COOH containing 0.1% (*w*/*v*) pepsin at a residue-to-solvent ratio of 1:20 (*w*/*v*) and the mixture was stirred for 24 h. Following filtration through a 540-mesh filter, the filtrate was salted out by addition of NaCl to a final concentration of 2.5 M in the presence of 0.05 M Tris-HCl (pH 7.0), and the solution was kept at 8 °C for 24 h. The resulting precipitate was subjected to centrifugation and dialysis in the same manner as previously described for ASC. The resulting dialysate was lyophilized, and is hereafter referred to as ‘‘pepsin-soluble collagen, PSC’’. The extraction yield of PSC was calculated as described above for ASC.

### 3.3. Preparation of the Electrospun Nanofibrous Patches

The spinning solutions for the fabrication of the various collagen/ulvan/PEO nanofibrous matrices were prepared in 0.5 M CH_3_COOH at room temperature under stirring for 24 h to ensure their homogeneity. ASCs and PSCs isolated from the scales of the different fish were dissolved at a concentration of 1% (*w*/*v*) followed by the addition of ulvan at 1% (*w*/*v*) and PEO at 2.5% (*w*/*v*), resulting in a 1:1:2.5 (*w*/*w*) collagen:ulvan:PEO ratio in the fabricated matrices. Electrospinning was performed using a γ-High Voltage Research DC power supply generator with a maximum voltage of 50 kV (Gamma High Voltage Research, Ormond Beach, FL, USA). The polymer solutions were loaded into 10 mL disposable syringes fitted with 23 G stainless steel blunt needle with the syringes mounted on a horizontally positioned programmable syringe pump Harvard PHD 2000 (Harvard Apparatus, Holliston, MA, USA). The solution feeding rate, applied voltage, and tip-to-collector distance were fixed at 1 mL/h, 27 kV, and 30 cm, respectively, and the electrospun nanofibers were deposited on aluminum foil wrapped on a RC-6000 rotating drum collector (NaBond Technologies, Hong Kong) at a rotation speed of 400 rpm. Temperature and relative humidity were 20 ± 2 °C and 60 ± 5%, respectively.

### 3.4. Scanning Electron Microscopy (SEM)

For SEM analysis, the lyophilized collagen samples and collagen-based nanofibers were initially precoated with a conductive layer of sputtered gold and examined using a PhenomWorld desktop scanning electron microscope (Thermo Fischer Scientific, Waltham, MA, USA) with tungsten filament (10 kV) and a charge reduction sample holder. Using the embedded image analysis software (Fibermetric/Phenom Pro Suite v.2.3, Eindhoven, The Netherlands), the diameters of 100 fibers from each SEM image were measured, and the average fiber diameter was determined.

### 3.5. Fourier Transform Infrared Spectroscopy (FT-IR)

FT-IR spectra of the lyophilized collagen samples and collagen-based nanofibers were measured on a Bruker Alpha II (Billerica, MA, USA) spectrometer using the attenuated total reflection (ATR) method at room temperature in the range of 400–4000 cm^−1^ with a resolution of 4 cm^−1^.

### 3.6. Ultraviolet-Visible Spectroscopy (UV-Vis)

UV-Vis spectra of ASC and PSC solutions were recorded on a Perkin Elmer Lambda 40 spectrophotometer (PerkinElmer Ltd., Buckinghamshire, UK) in the range of 200–400 nm. The samples were prepared by dissolving the collagen samples in 0.5 M CH_3_COOH with a sample-to-solution ratio of 1:1000 (*w/v*).

### 3.7. X-ray Diffraction (XRD)

XRD analysis of freeze-dried ASCs and PSCs was performed on a Bruker D8 Advance diffractometer (Bruker, Karlsruhe, Germany), which was operated at a generator voltage of 40 kV and a current of 25 mA. The X-ray source is CuKα radiation (λ = 0.15408 nm). The diffraction patterns were collected at a scanning speed of 0.220 sec/step within a 2*θ* scanning range of 3° to 55°.

### 3.8. Thermogravimetric Analysis (TGA)

Thermogravimetric analysis of the isolated collagens was performed using a TA Thermogravimetric Analyzer (TGA 55, TA instruments, New Castle, DE, USA) under nitrogen flow of 25 mL/min at a heating rate of 10 °C/min in the range 40–700 °C. Sample temperature, sample weight, and heat flow were recorded continuously.

### 3.9. Sodium Dodecyl Sulfate–Polyacrylamide Gel Electrophoresis (SDS–PAGE)

Protein patterns of collagen were determined using SDS–PAGE according to the method of Laemmli (1970) [75]. Collagenous solutions were first dissolved at 4 mg/mL in 20 mM CH_3_COOH, then mixed at a ratio 1:1 (*v*/*v*) with the sample buffer [0.25 M Tris–HCl, pH 6.8, containing 4% (*w*/*v*) SDS, 20% (*v*/*v*) glycerol, and 0.2% (*w*/*v*) bromophenol blue] in the presence of 10% (*v*/*v*) 2-mercaptoethanol and finally boiled for 10 min. Each sample (20 μg) was loaded onto the polyacrylamide gel consisting of a 7% running gel and a 3% stacking gel and subjected to electrophoresis at a constant current of 14 mA/gel using a mini-vertical electrophoresis system (Mini-PROTEAN System, Bio-Rad Laboratories, Hercules, CA, USA) [59]. Gels were stained for 1 h using 0.25% (*w*/*v*) Coomassie brilliant blue R250 dissolved in MeOH-H_2_O-CH_3_COOH (5:4:1, *v*/*v*/*v*) and subsequently destained with 40% (*v*/*v*) methanol and 10% (*v/v*) CH_3_COOH. Regular MW markers (10–180 kDa, BlueStar Prestained Protein Marker, Nippon Genetics Europe GmbH, Düren, Germany) were used to estimate the MW of proteins. Quantification of band intensity was determined using ImageJ software (NIH, Bethesda, MD, USA).

### 3.10. Determination of Hydroxyproline Content

Initially, the collagen samples were hydrolyzed for 3 h at 120 °C in 6 N HCl. Hydroxyproline content was determined in the hydrolysate using a colorimetric method employing Ehrlich’s reagent according to the manufacturer’s instructions. Absorbance was measured at 560 nm using an Infinite M200PRO plate reader (TECAN, Männedorf, Switzerland). Hydroxyproline standard solutions with concentrations ranging from 0.2 to 1 μg were used for the standard curve preparation. Hydroxyproline content was calculated and expressed as mg/g sample.

## 4. Conclusions

In the present study, the collagenous content of the scales from five commercially important Mediterranean fish species (*E. marginatus*, *M. barbatus*, *P. pagrus*, *S. aurata*, and *U. cirrosa*), as well as from the Atlantic salmon (*S. salar*), was comprehensively examined. Acid- and pepsin-soluble collagens were isolated from the scales of these six species, with the yield of PSCs being higher than that for ASCs (1.4 to 4 times), indicating that the collagen’s telopeptide region is highly cross-linked in fish scales. All collagens were characterized as type I collagen and maintained their triple-helical structure after isolation. SEM observations confirmed the characteristic fibrous structures, while FT-IR spectroscopic analysis verified the characteristic absorption bands for collagen proteins. Both ASCs and PSCs, blended with ulvan and PEO, formed homogenous solutions that were electrospun towards the fabrication of uniform, bead-free fibers. All the prepared fibrous scaffolds were of similar randomly oriented morphology, forming a fibrous network of smooth cylindrical fibers sizing in the nanometer scale. Our results suggest that ASCs and PSCs isolated from fish scales can be considered as alternative collagenous material for health-related applications in the biomedical sector. It is important to note that even though fish scales are presently a waste material of the fish processing and canning industry and fish markets with no value, they could easily be exploited for the production of high added-value biomaterials. The fact that the collagen obtained from scales of different fish species does not exhibit significant differences in its properties would allow for their concurrent commercial processing towards the isolation of collagen in a sustainable and economically viable way.

## Figures and Tables

**Table 1:** Collagen yield (*w*/*w* %) and hydroxyproline content (mg/g) of ASCs and PSCs isolated from fish scales.

Species	ASC		PSC	
	Yield (% Dry Weight) ^1^	Hydroxyproline Content (mg/g) ^2^	Yield (% Dry Weight) ^1^	Hydroxyproline Content (mg/g) ^2^
*Epinephelus marginatus*	0.39 ± 0.02	46.9 ± 3.5	1.54 ± 0.06	56.7 ± 1.9 ^a^
*Mullus barbatus*	0.62 ± 0.06	52.5 ± 6.5 ^a,b^	1.04 ± 0.04	58.6 ± 4.8 ^b,c^
*Pagrus pagrus*	0.61 ± 0.05	42.9 ± 4.5	1.32 ± 0.06	50.6 ± 1.5 ^d^
*Sparus aurata*	1.13 ± 0.06	46.6 ± 6.8	1.57 ± 0.06	39.6 ± 3.4 ^a,b,d,e^
*Umbrina cirrosa*	0.41 ± 0.02	38.3 ± 5.3 ^b^	1.61 ± 0.07	47.0 ± 3.8 ^c,f^
*Salmo salar*	0.87 ± 0.02	35.1 ± 3.1 ^a^	2.03 ±0.10	58.4 ± 8.3 ^e,f^

^1^ Yield is based on dry weight of scales. Data are presented as the percent of scales dry weight. Data are expressed as mean ± SD (*n* = 3). ^2^ Data are presented as mg of hydroxyproline per g of ASCs or PSCs dry weight. Hydroxyproline content values are expressed as mean ± SD (*n* = 4). Identical superscripts in the same column denote statistically significant differences (*p* < 0.05).

**Table 2:** Range of diameter and average diameter of the fabricated collagen-based nanofibers.

Species	ASC		PSC	
	Range of Diameter (nm)	Average Diameter (nm)	Range of Diameter (nm)	Average Diameter (nm)
*Epinephelus marginatus*	121–593	332 ± 65	54–564	305 ± 66
*Mullus barbatus*	118–637	348 ± 79	90–800	364 ± 74
*Pagrus pagrus*	128–624	287 ± 51	167–624	307 ± 66
*Sparus aurata*	184–497	314 ± 71	104–595	330 ± 70
*Umbrina cirrosa*	115–590	320 ± 65	115–492	273 ± 48
*Salmo salar*	120–553	297 ± 65	170–592	305 ± 57

## Data Availability

The data that support the findings of this study are available from the corresponding authors upon request.

## Supplementary Materials

The following supporting information can be downloaded at: https://www.mdpi.com/article/10.3390/md20110664/s1, Figure S1: UV spectra of ASC and PSC isolated from the scales of *E. marginatus*; Figure S2: UV spectra of ASC and PSC isolated from the scales of *M. barbatus*; Figure S3: UV spectra of ASC and PSC isolated from the scales of *P. pagrus*; Figure S4: UV spectra of ASC and PSC isolated from the scales of *S. aurata*; Figure S5: UV spectra of ASC and PSC isolated from the scales of *U. cirrosa*; Figure S6: UV spectra of ASC and PSC isolated from the scales of *S. salar*; Figure S7: X-ray diffraction diagrams of ASC and PSC from *U. cirrosa*, indicating a highly ordered structure in the case of ASC; Figure S8: DTG thermogravimetric curves of ASCs and PSCs isolated from (A) *E. marginatus*, (B) *M. barbatus*, (C) *P. pagrus*, (D) *S. aurata*, (E) *U. cirrosa* and (F) *S. salar*; Table S1: Absorption bands observed in the FT-IR spectra of ASCs and PSCs isolated from the fish scales and their assignment.
