# Supplementary material for: Valorization of Fish Waste: Isolation and Characterization of Acid- and Pepsin-Soluble Collagen from the Scales of Mediterranean Fish and Fabrication of Collagen-Based Nanofibrous Scaffolds
Source: Mar Drugs. 2022 Oct 25;20(11):664. doi: 10.3390/md20110664 (PMC9697972; doi:10.3390/md20110664)
Supplement: Supplementary file 1 [file marinedrugs-20-00664-s001.zip › marinedrugs-1963964-supplementary.pdf]

Supplementary materials for

Valorization of Fish Waste: Isolation and Characterization of Acid- and Pepsin-soluble Collagen from the Scales of Mediterranean Fish and Fabrication of Collagen-based Nanofibrous Scaffolds

Leto-Aikaterini Tziveleka [1], Stefanos Kikionis [1], Labros Karkatzoulis [1,2], Kostas Bethanis [2], Vassilios Roussis [1] and Efstathia Ioannou [1,*]

[1] Section of Pharmacognosy and Chemistry of Natural Products, Department of Pharmacy, National and Kapodistrian University of Athens, Panepistimiopolis Zografou, Athens 15771, Greece

[2] Laboratory of Physics, Department of Biotechnology, Agricultural University of Athens, Athens 11855, Greece

* Correspondence: eioannou@pharm.uoa.gr

Table S1. Absorption bands observed in the FT-IR spectra of ASCs and PSCs isolated from the fish scales and their assignment.

Region	Wavenumber (cm^{-1})											
	E. marginatus		*M. barbatus*		*P. pagrus*		*S. aurata*		*U. cirrosa*		*S. salar*	
	**ASC**	**PSC**	**ASC**	**PSC**	**ASC**	**PSC**	**ASC**	**PSC**	**ASC**	**PSC**	**ASC**	**PSC**
Amide A	3295	3287	3293	3289	3293	3283	3285	3283	3293	3283	3291	3293
Amide B	2920	2918	2931	2931	2933	2931	2931	2929	2931	2933	2933	2933
Amide I	1644/ 1633	1633	1635	1633	1633	1633	1633	1633	1633	1631	1644/ 1635	1631
Amide II	1541	1538	1539	1538	1539	1538	1538	1538	1539	1539	1539	1532
CH_2 bend	1448	1448	1450	1448	1450	1448	1448	1448	1448	1446	1448	1446
Amide III	1236	1234	1234	1234	1236	1234	1234	1234	1234	1234	1234	1232

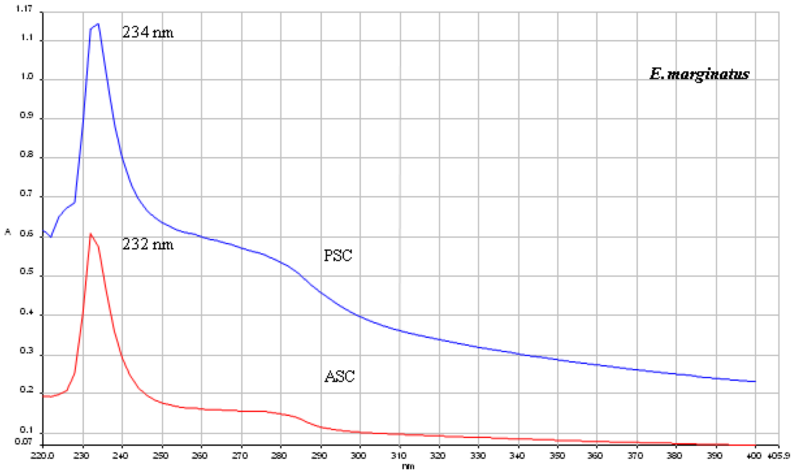

Figure S1. UV spectra of ASC and PSC isolated from the scales of *E. marginatus*,

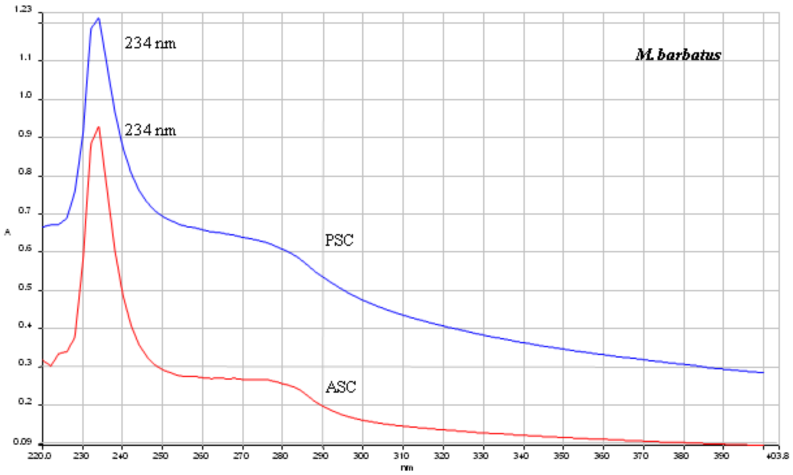

Figure S2. UV spectra of ASC and PSC isolated from the scales of *M. barbatus*.

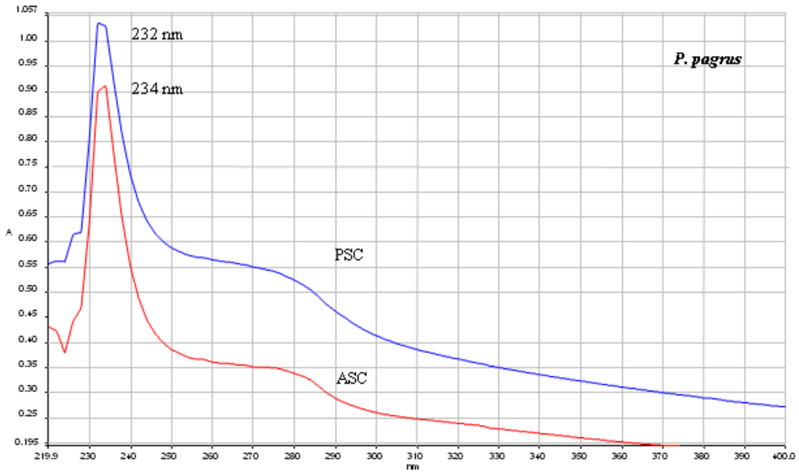

Figure S3. UV spectra of ASC and PSC isolated from the scales of *P. pagrus*.

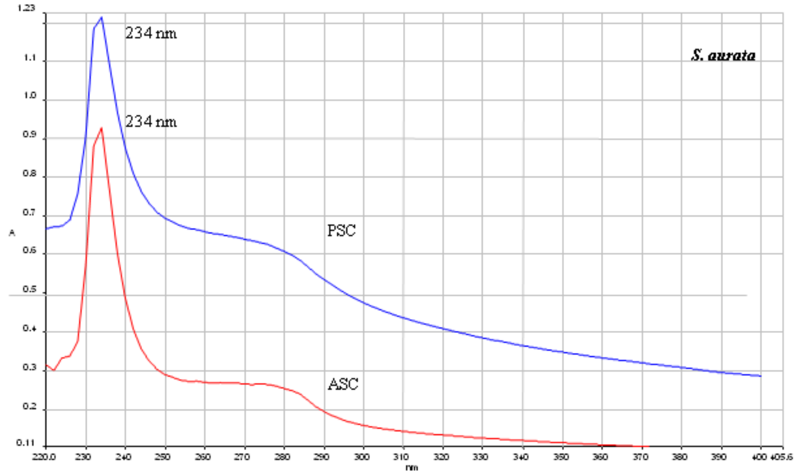

Figure S4. UV spectra of ASC and PSC isolated from the scales of *S. aurata*.

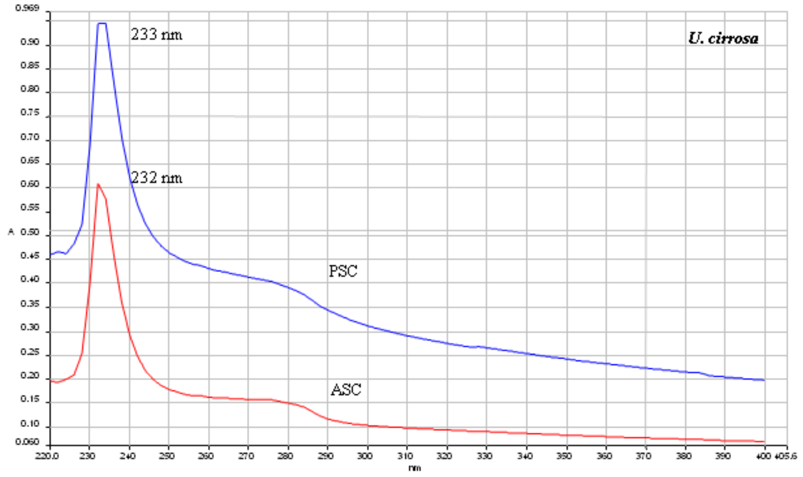

Figure S5. UV spectra of ASC and PSC isolated from the scales of *U. cirrosa*.

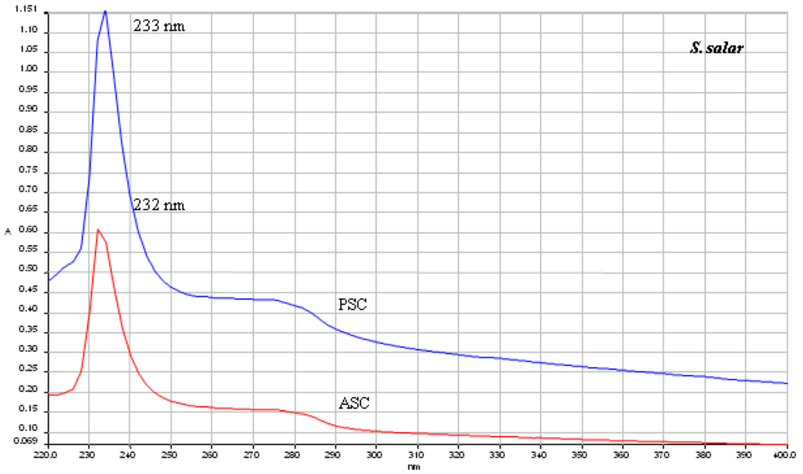

Figure S6. UV spectra of ASC and PSC isolated from the scales of *S. salar*.

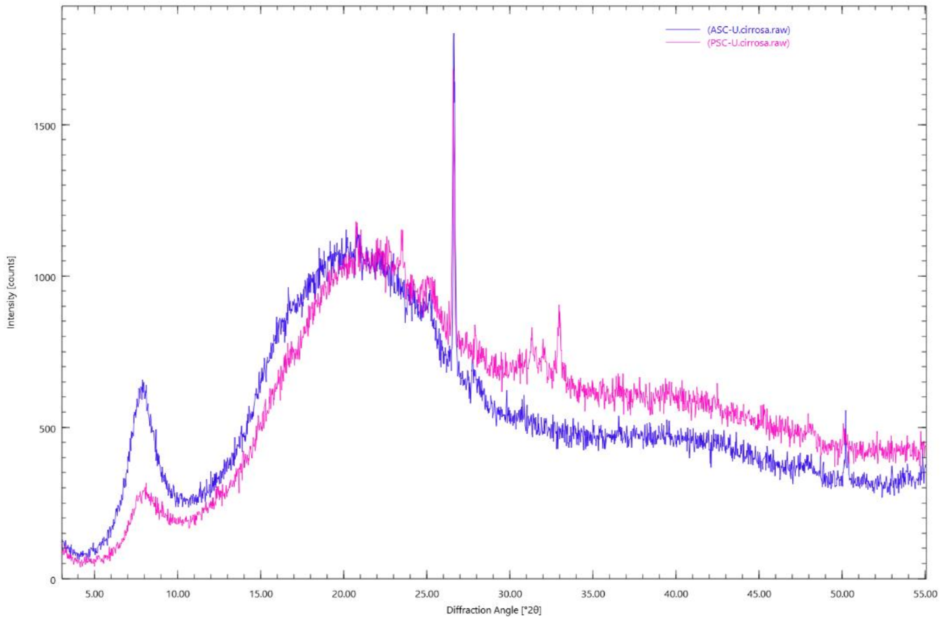

Figure S7. X-ray diffraction diagrams of ASC and PSC from *U. cirrosa*, indicating a highly ordered structure in the case of ASC.

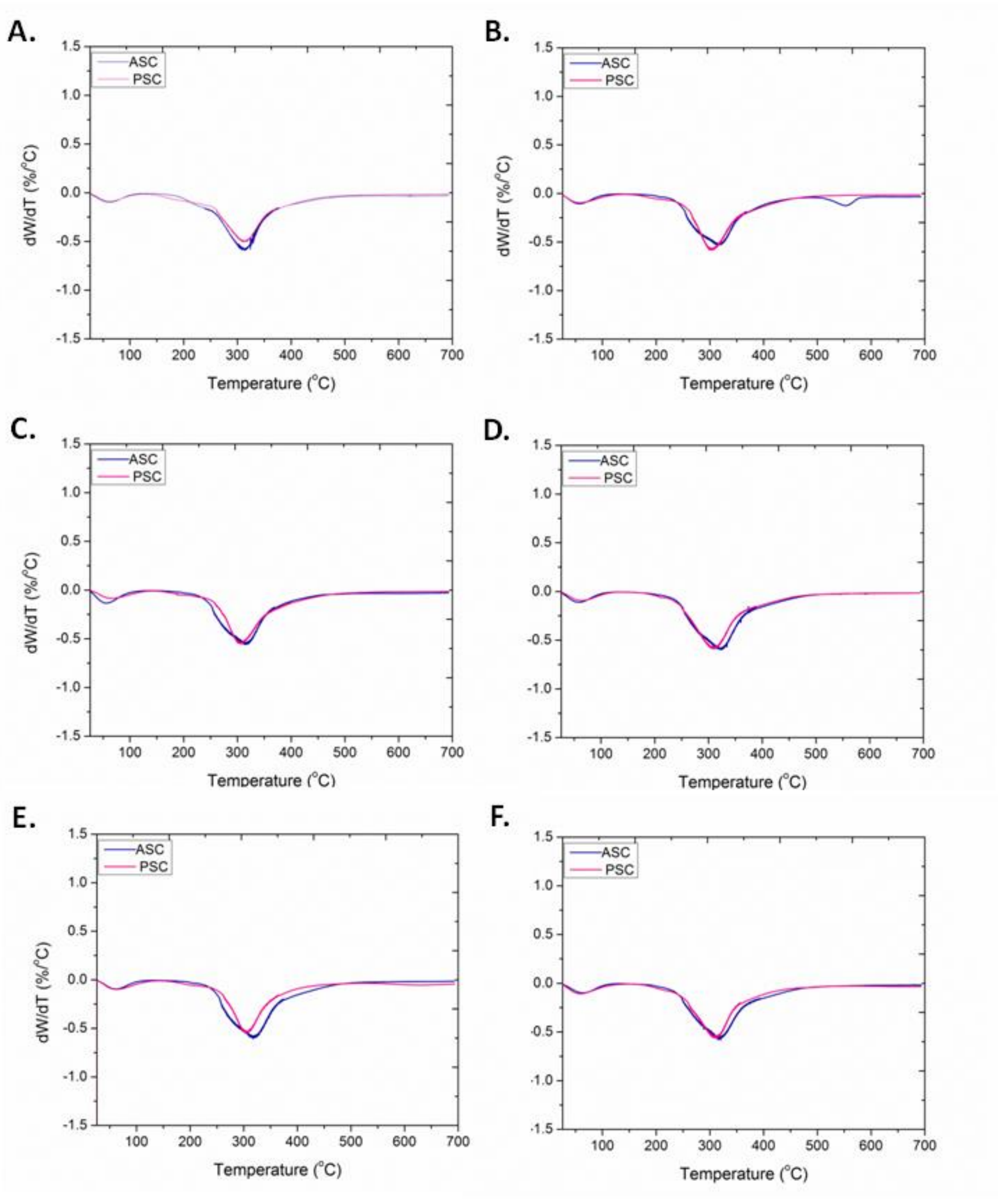

Figure S8. DTG thermogravimetric curves of ASCs and PSCs isolated from (**A**) *E. marginatus*, (**B**) *M. barbatus*, (**C**) *P. pagrus*, (**D**) *S. aurata*, (**E**) *U. cirrosa* and (**F**) *S. salar*.
